# A combined analysis of bulk and single-cell sequencing data reveals that depleted extracellular matrix and enhanced immune processes co-contribute to fluorouracil beneficial responses in gastric cancer

**DOI:** 10.3389/fimmu.2022.999551

**Published:** 2022-09-15

**Authors:** Shaowei Dong, Siyu Zhang, Pan Zhao, Guanchuan Lin, Xiaoshi Ma, Jing Xu, Hao Zhang, Jiliang Hu, Chang Zou

**Affiliations:** ^1^The Second Clinical Medical College, The First Affiliated Hospital of Southern University of Science and Technology, Jinan University (Shenzhen People’s Hospital), Shenzhen, China; ^2^Integrated Chinese and Western Medicine Postdoctoral Research Station, Jinan University, Guangzhou, China; ^3^School of Medicine, Life and Health Sciences, The Chinese University of Hong Kong (Shenzhen), Shenzhen, China; ^4^School of Medicine, The First Affiliated Hospital, Southern University of Science and Technology, Shenzhen, China; ^5^Department of Biochemistry and Molecular Biology, School of Basic Medical Sciences, Southern Medical University, Guangzhou, China; ^6^Guangdong Provincial Key Laboratory of Single Cell Technology and Application, Southern Medical University, Guangzhou, China; ^7^Institute of Precision Cancer Medicine and Pathology, Jinan University Medical College, Guangzhou, China; ^8^Guangdong Engineering Technological Research Center for Nervous Anatomy and Related Clinical Applications, Shenzhen, China

**Keywords:** fluorouracil response, gastric cancer, tumor microenvironments, extracellular matrix, immune components

## Abstract

Fluorouracil, also known as 5-FU, is one of the most commonly used chemotherapy drugs in the treatment of advanced gastric cancer (GC). Whereas, the presence of innate or acquired resistance largely limits its survival benefit in GC patients. Although accumulated studies have demonstrated the involvement of tumor microenvironments (TMEs) in chemo-resistance induction, so far little is known about the relevance of GC TMEs in 5-FU resistance. To this end, in this study, we investigated the relationship between TME features and 5-FU responses in GC patients using a combined analysis involving both bulk sequencing data from the TCGA database and single-cell RNA sequencing data from the GEO database. We found that depleted extracellular matrix (ECM) components such as capillary/stroma cells and enhanced immune processes such as increased number of M1 polarized macrophages/Memory T cells/Natural Killer T cells/B cells and decreased number of regulatory T cells are two important features relating to 5-FU beneficial responses in GC patients, especially in diffuse-type patients. We further validated these two features in the tumor tissues of 5-FU-benefit GC patients using immunofluorescence staining experiments. Based on this finding, we also established a Pro (63 genes) and Con (199 genes) gene cohort that could predict 5-FU responses in GC with an AUC (area under curve) score of 0.90 in diffuse-type GC patients, and further proved the partial applicability of this gene panel pan-cancer-wide. Moreover, we identified possible communications mediated by heparanase and galectin-1 which could regulate ECM remodeling and tumor immune microenvironment (TIME) reshaping. Altogether, these findings deciphered the relationship between GC TMEs and 5-FU resistance for the first time, as well as provided potential therapeutic targets and predicting rationale to overcome this chemo-resistance, which could shed some light on developing novel precision treatment strategies in clinical practice.

## Introduction

Gastric cancer (GC) represents one of the most common types of cancer and accounts for 5.6% of newly diagnosed cancer cases. In 2020, GC caused more than 70,000 deaths worldwide (7.7% and ranked 3^rd^) (1). 5-FU is one of the most commonly used antimetabolite drugs. 5-FU monotherapy, or in combination with other therapeutics, has been suggested as the standard regimen for advanced GC treatment in many countries (2, 3). Unfortunately, the 5-year survival rate of patients with advanced GC is only 10-15%, and due to the acquired chemoresistance, many GC patients still suffer from recurrence and metastasis after an initial response to 5-FU-based chemotherapy.

In the human body, 5-FU functions as a thymidylate synthase (TS) inhibitor. After being taken, 5-FU is first converted to fluorodeoxyuridine monophosphate (FdUMP), which can form a stable complex with TS. TS functions by catalyzing the conversion of deoxyuridine monophosphate (dUMP) to deoxythymidine monophosphate (dTMP). After binding to FdUMP, this process is inhibited, thus causing cytotoxicity (4, 5). Traditional chemoresistance explorations mainly focused on 5-FU pathway-related enzymes (6, 7) or ATP-binding cassette (ABC) transporter-mediated drug efflux process (8–10), and few attempts have been made in the investigations of the relationships between TMEs and chemoresistance due to the lack of means in the exploration of TMEs at a cellular level.

A tumor is a complex mixture of different cell types, and many of them have been reported of chemoresistance relevance, such as heterogeneity of cancer cells, stiffness of ECM, depletion of immune cells, and enrichment of tumor-suppressive immune cells including M2 polarized macrophages, regulatory T cells, and B cells (11). Recent advances in single-cell RNA sequencing (scRNAseq) technologies have made it possible to examine the gene expression profiles within a single cell while uncovering abnormal communications between different cell types (12, 13), which greatly facilitates the exploration of TME-involved chemoresistance. In this study, using a combined analysis of scRNAseq data and bulk sequencing data, we provided evidence that depleted ECM and enriched immune components are two important TME features leading to 5-FU beneficial responses. We further established a Pro and Con gene cohort that could predict 5-FU responses in GC patients. Moreover, we identified key regulatory communications responsible for different 5-FU responses. This study provides another perspective on the exploration of chemoresistance.

## Materials and methods

### TCGA data retrieval

Phenotype information of 7,773 TCGA samples covering 18 different cancer types was downloaded from GDC portal (genomic data commons data portal, www.portal.gdc.cancer.gov). Only tumor samples were involved in this study (“sample_type.samples” = “Primary Tumor”). Survival information was determined using “days_to_death.diagnoses” and “days_to_last_follow_up.diagnoses” information from phenotype files. Normalized gene expression data of involved TCGA samples were downloaded from RNAseqDB (14).

### Survival analysis

Survival analysis was performed using R package “survival” and “survminer”, and Kaplan-Meier survival plot was generated using survfit() function. In the screening of Pro and Con genes, gene expression values of each input gene were classified into either “high” or “low” categories using a median value as classifier, and a Pvalue < 0.01 was used as a significant cut-off. A Pro gene was determined as “high” expression beneficial for survival probability and a Con gene was determined as “low” expression beneficial for survival probability.

### GO enrichment analysis

GO enrichment analysis of Pro and Con genes was performed using R package “clusterProfiler” and “org.Hs.eg.db”. All input genes were first transformed into “ENTREZID” before the enrichment analysis, and a “Benjamini-Hochberg” method was used in generating adjusted pvalues. A P.adjust value of 0.05 was used as a significance cut-off.

### ROC analysis

ROC analysis was performed using R package “ROCR” and “pROC”, and the AUC values (area under curves) were calculated using auc() function.

### scRNAseq data analysis

scRNAseq raw count files were downloaded from GEO online database under accession number GSE183904. A “doublet detection” process was performed on each sample separately using R package “DoubletFinder” (15) using 7.5% as duplet cutoff, and all doublets were filtered prior to the integration process.

Integration was performed through R package “Seurat” (16) using FindIntegrationAnchors() and IntegrateData() functions. After a quality control process, all cells with 200-2,500 unique feature counts and less than 15% of mitochondrial counts were retained. The combined data was scaled using ScaleData() function followed by a linear dimensional reduction process using RunPCA() function and a non-linear dimensional reduction process using UMAP (Uniform Manifold Approximation and Projection) method (dims = 1:25, resolution = 1).

### The decomposition process of TCGA samples

The decomposition process was performed using R packages “Biobase” and “BisqueRNA” (17). @metadata and @assays$RNA@data were used as scRNAseq data input, and normalized TCGA expression file was used as bulk sequencing input.

### Immunostaining

Patient tumor sections were obtained from the Pathology department of Shenzhen People’s Hospital, and all the sections were incubated in 3% H_2_O_2_ at room temperature for 10min prior to further antibody incubation. The following primary antibodies were used in this study (CD206: CST 91992S; CD79A: ABClonal A0331; HPSE: ABClonal A5727; TAGLN: ABClonal A21209; COL1A2: ZENBIO 343277; LGALS1: ABClonal A1580). After an incubation of 2h at room temperature with primary antibody, the sections were further incubated with goat anti-rabbit IgG H&L Cy5 secondary antibody (Abcam ab6564) and different stains (green: Alexa Fluor™ 488 Tyramide, Invitrogen B40922; red: Alexa Fluor™ 555 Tyramide, Invitrogen B40923; purple, Alexa FluorTM 647 Tyramide, Invitrogen B40926).

### Ethical considerations

This study was approved by the ethical committee of Shenzhen People’s Hospital. All participating patients have provided written consent.

## Results

### Favorable prognosis effects of 5-FU treatment on diffuse-type GC patients

**Figure 1:**
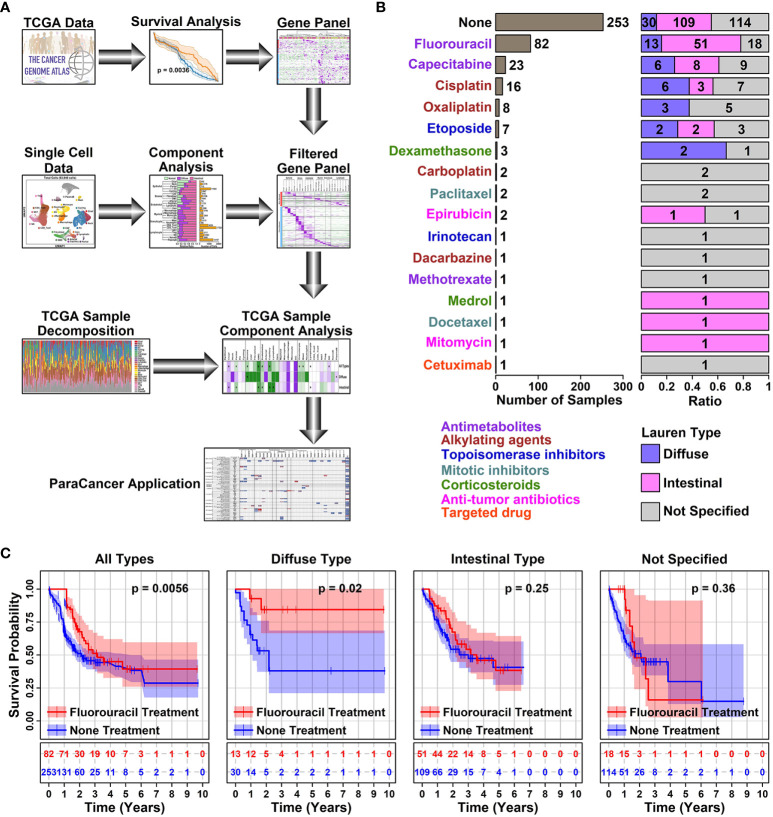
5-FU responses in different subtypes of STAD samples. **(A)** Schematic diagram illustrating the research design of this study. In this study, a Pro and Con gene panel was first screened using TCGA data, and the expression status of these genes was further examined in different cell types of GC samples using scRNAseq data. inally, the conclusions of this study were validated using decomposited TCGA data; **(B)** Bar plot showing the number of samples with a certain type of drug treatment. All the STAD sample information was retrieved from TCGA online database; **(C)** Plots showing the survival status of patients with or without 5-FU treatment. The survival information of involved STAD samples was retrieved from the TCGA database, and a Kaplan-Meier plotter was used to estimate the survival information. A *p* < 0.05 was used as a significance cutoff.

The rationale of this study is to investigate the relationship between 5-FU responses in GC patients and their TMEs features. A simplified flowchart illustrating the research design of this study is listed in **Figure 1A**.

Among 407 TCGA STAD (Stomach adenocarcinoma) tumor samples, 253 of them have no records of drug treatments, 106 of them have antimetabolite treatment (Fluorouracil or 5-FU, 82; Capecitabine, 1; Methotrexate, 1); 25 of them have alkylating agent treatment (Cisplatin, 16; Oxaliplatin, 8; Dacarbazine, 1); 8 of them have topoisomerase inhibitor treatment (Etoposide, 7; Irinotecan, 1); 3 of them have mitotic inhibitor treatment (Paclitaxel, 2; Docetaxel, 1); 4 of them have corticosteroid treatment (Dexamethasone, 3; Medrol, 1); 3 of them have anti-tumor antibiotic treatment (Epirubicin, 2; Mitomycin, 1); 1 of them has targeted drug treatment (Cetuximab, 1), as listed in **Figure 1B** and detailed in **Supplementary Table 1**. Among all these treatments, 5-FU is the only drug with >=10 treated samples across all different Lauren subtypes of GC (Diffuse type, 13; Intestinal type, 51; Not specified, 18), hence we use this drug in the latter analysis.

5-FU chemotherapy is one of the prior choices for advanced GC treatment, and in our analysis, samples treated with 5-FU did show a significant increase in survival rate compared to samples receiving no drug treatment (1 year, 85.59% vs 51.78%, ***; 3 years, 23.17% vs 9.88%, **), as summarized in **Table 1**. Among different subtypes of GC samples with 5-FU treatment, diffuse samples have a higher survival rate compared to intestinal samples (1-year survival rate, 92.31% vs 86.27%; 3-year survival rate, 30.77% vs 27.45%). A further prognosis analysis also shows that 5-FU responses vary among different GC subtypes, as demonstrated in **Figure 1C**: 5-FU treatment has a favorable prognosis effect on all GC samples (*p* = 0.0056) and diffuse samples (*p* = 0.02), and this favorable prognosis effect is not significant on intestinal samples (*p* = 0.25), nor unspecified samples (*p* = 0.36), suggesting 5-FU might be a better chemotherapy solution in treating diffuse GC patients.

**Table 1:** Survival times of STAD samples from TCGA online database.

		All Types		Diffuse Type		Intestinal Type		Not Specified	
		5-FU	None	5-FU	None	5-FU	None	5-FU	None
	Total	82	253	13	30	51	109	18	114
1 Year	Death	11	122	1	16	7	43	3	63
	Alive	71	131	12	14	44	66	15	51
	Survival	86.59%	51.78%	92.31%	46.68%	86.27%	60.55%	83.33%	44.74%
	*X*^2^	29.90		6.11		9.54		7.78	
	*P* value	***		*		***		**	
3 Year	Death	63	228	9	28	37	94	17	106
	Alive	19	25	4	2	14	15	1	8
	Survival	23.17%	9.88%	30.77%	6.67%	27.45%	13.76%	5.55%	7.02%
	*X*^2^	8.46		2.61		3.51		0.45	
	*P* value	**		>0.05		>0.05		>0.05	
5 Year	Death	75	245	12	28	46	105	17	112
	Alive	7	8	1	2	5	4	1	2
	Survival	8.53%	3.16%	7.69%	6.67%	9.80%	3.67%	5.56%	1.75%
	*X*^2^	3.02		0.00		1.44		0.02	
	*P* value	>0.05		>0.05		>0.05		>0.05	

***p < 0.001; **p < 0.05; *p < 0.1.

### A pro and con gene cohort in GC 5-FU-response prediction

**Figure 2:**
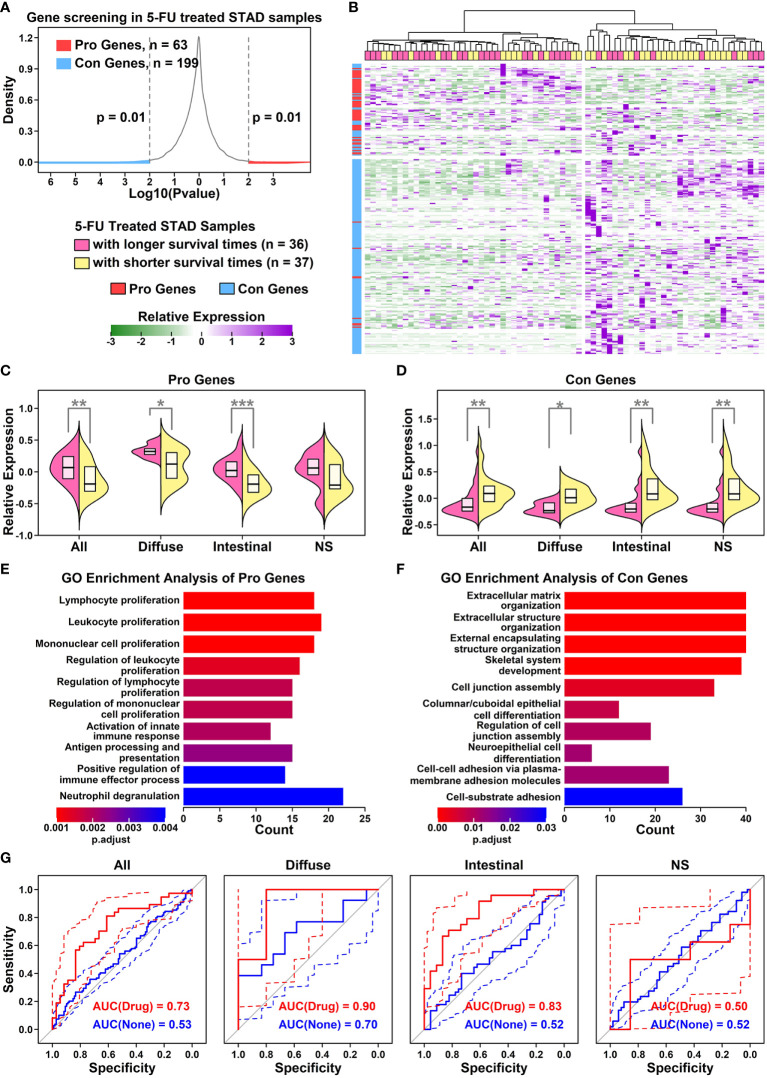
Establishment of a Pro and Con gene cohort **(A)** Density plot showing the *p* value distribution of genes in the survival analysis of 5-FU treated STAD samples. x-axis represents the log10 *p* value of each input gene in Kaplan-Meier analysis, and y-axis represents the density distributions of all *p* values; **(B)** Heatmap representing relative expression values of Pro (red) and Con (blue) genes across all 5-FU treated STAD samples. Pink represents 5-FU treated STAD samples with longer survival times (> median), and yellow represents 5-FU treated STAD samples with shorter survival times (<= median); **(C)** Dual violin plot showing the relative expression values of Pro gene cohort across samples of different GC subtypes (*p < 0.05; **p < 0.01; ***p < 0.001. Two-tailed t-test); **(D)** Dual violin plot showing the relative expression values of Con gene cohort across samples of different GC subtypes. A two-tailed t-test was used(*p < 0.05; **p < 0.01; ***p < 0.001. Two-tailed t-test); **(E)** Top 10 enriched GO terms of Pro genes; **(F)** Top 10 enriched GO terms of Con genes; **(G)** ROC curves representing the separation ability of Pro and Con gene cohort in separating different subtypes of 5-FU-benefit samples from 5-FU-futile samples.

To screen for genes involved in 5-FU responses, we retrieved normalized gene expression data of 5-FU-treated STAD samples from RNAseqDB (14). The expression status of each protein-coding gene (“High” or “Low” in comparison to median value) and survival times/status of related STAD samples were used in the prognosis analysis. 63 Pro genes (higher expression beneficial for 5-FU treated STAD samples with longer survival times, n = 36, *p* < 0.01) and 199 Con genes (higher expression beneficial for 5-FU treated STAD samples with shorter survival times, n = 37, *p* < 0.01), as shown in **Figure 2A** and listed in **Supplementary Table 2**. Most of the Pro genes (red) have relatively higher expression values in 5-FU treated STAD samples with longer survival times (top-left panel in **Figures 2B, C**), and most of the Con genes (blue) have relatively higher expression values in 5-FU treated STAD samples with shorter survival times (bottom-right panel in **Figures 2B, D**). Specifically, 5-FU-treated diffuse STAD samples with longer survival times have the highest expression values of Pro genes and lowest expression values of Con genes compared to these in other Lauren subtypes of STAD samples, indicating these genes might be related to a 5-FU beneficial effect. 5-FU treated STAD samples with longer survival times are renamed as 5-FU-benefit samples, and 5-FU treated STAD samples with shorter survival times are renamed as 5-FU-futile samples, hereafter.

GO enrichment results of Pro and Con genes are listed in **Figure 2E**. Among 63 Pro genes, the top enriched GO functions are immune-related items such as “Lymphocyte proliferation”, “Leukocyte proliferation” and “Mononuclear cell proliferation”, suggesting an immune infiltration process or enhanced immunity occurring in 5-FU-benefit samples. Among 199 Con genes, the top enriched GO functions are extracellular matrix (ECM) related items such as “ECM organization”, “Extracellular structure organization”, and “External encapsulating structure organization”, suggesting an ECM remodeling process in 5-FU-futile samples.

To evaluate whether these Pro and Con genes could be used to predict 5-FU responses in STAD samples, we use a ROC analysis to examine the separation ability of these genes (ZSCORE_Pro_ – ZSCORE_Con_) in separating 5-FU-benefit samples from 5-FU-futile samples (**Figure 2G**). Overall, this gene panel has the highest separation ability of 0.90 on 5-FU treated diffuse GC samples, 0.83 on 5-FU treated intestinal GC samples, 0.73 on all types of GC samples, and 0.50 on not specified GC samples. These AUC results are consistent with previous prognosis results, suggesting that these genes play an important role in the 5-FU chemotherapy process.

### Performance of the pro and con gene cohort in pan-cancer-wide drug-response prediction

**Figure 3:**
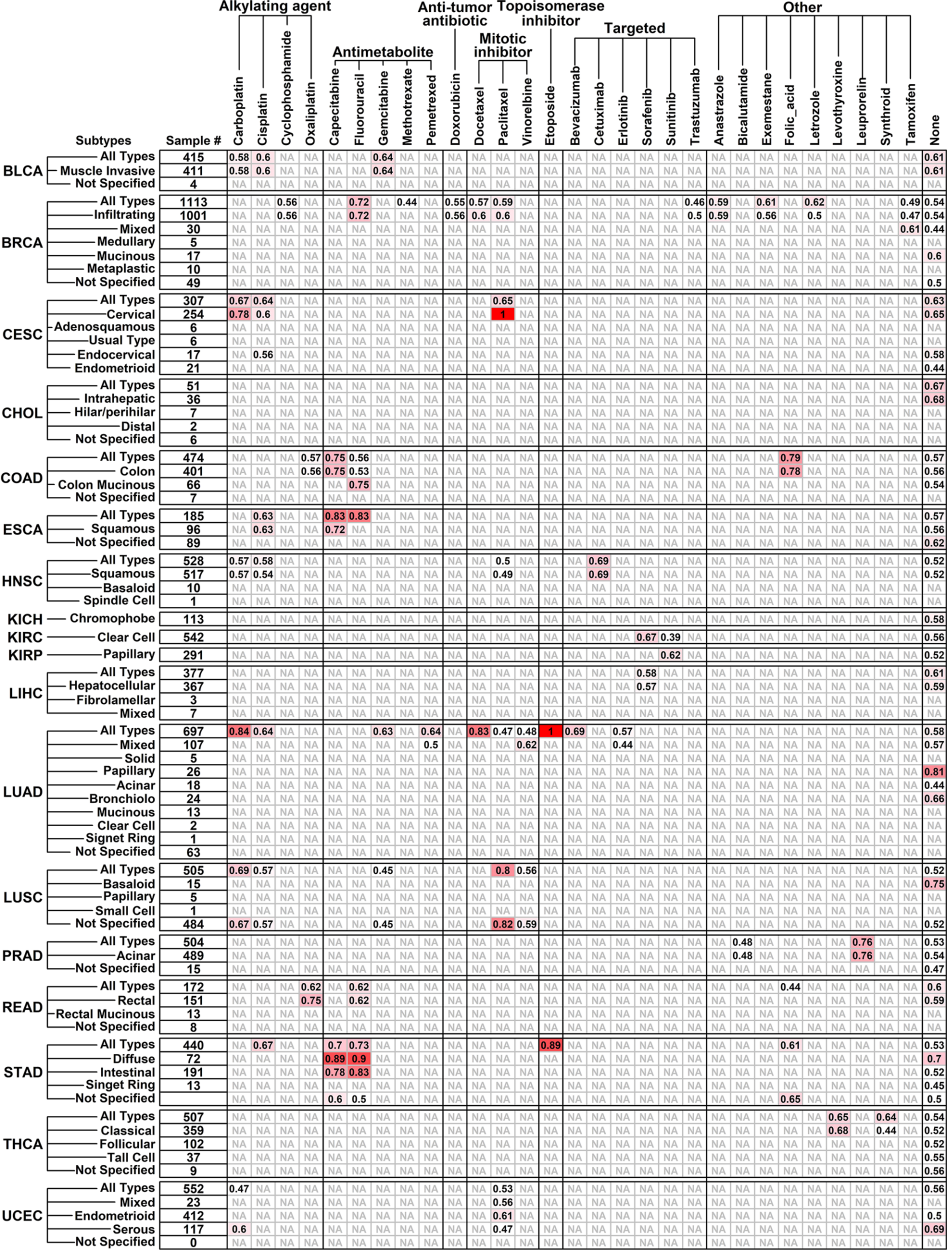
Performance of Pro and Con gene cohort in predicting different drug responses pan-cancer-wide. Tumor types/subtypes are listed on the left, and involved chemotherapy drugs are listed on top. AUC scores in separating drug-benefit samples from drug-futile samples under each drug/cancer combination are listed, and an “NA” represents “not applicable” due to small sample size (<6).

To evaluate the ability of the Pro and Con gene cohort in predicting responses of different chemotherapy drugs pan-cancer-wide, we retrieved the clinical information of 7,773 samples from TCGA online database involving 18 different cancer types and 75 drugs (**Supplementary Table 3**) together with their corresponding gene expression files from RNAseqDB (14), and calculated the AUC scores of gene cohort in separating specific-drug-treated samples of certain cancer type (subtype) with longer survival times from ones with shorter survival times. All the AUC results are detailed in **Figure 3**.

The Pro and Con gene cohort has a satisfactory prediction ability (AUC score >= 0.8) on 12 cancer/drug combinations, including Carboplatin on LUAD (lung adenocarcinoma, all types, 0.84); Capecitabine on ESCA (esophageal carcinoma, all types, 0.83) and STAD (diffuse type, 0.89); Fluorouracil on ESCA (all types, 0.83) and STAD (diffuse type, 0.90; intestinal 0.83); Docetaxel on LUAD (all types, 0.83); paclitaxel on CESC (cervical squamous cell carcinoma and endocervical adenocarcinoma, cervical type, 1.00) and LUSC (lung squamous cell carcinoma, all types, 0.80; not specified, 0.82); Etoposide on LUAD (all types, 1.00) and STAD (all types, 0.89). Interestingly, the Pro and Con gene panel has a similar prediction ability on Capecitabine and Fluorouracil, which is not surprising because Capecitabine is used as a replacement for 5-FU in many cancer treatments (18), and this result further validated the robustness of our results.

### Single-cell transcriptome analysis of GC diffuse and intestinal samples

**Figure 4:**
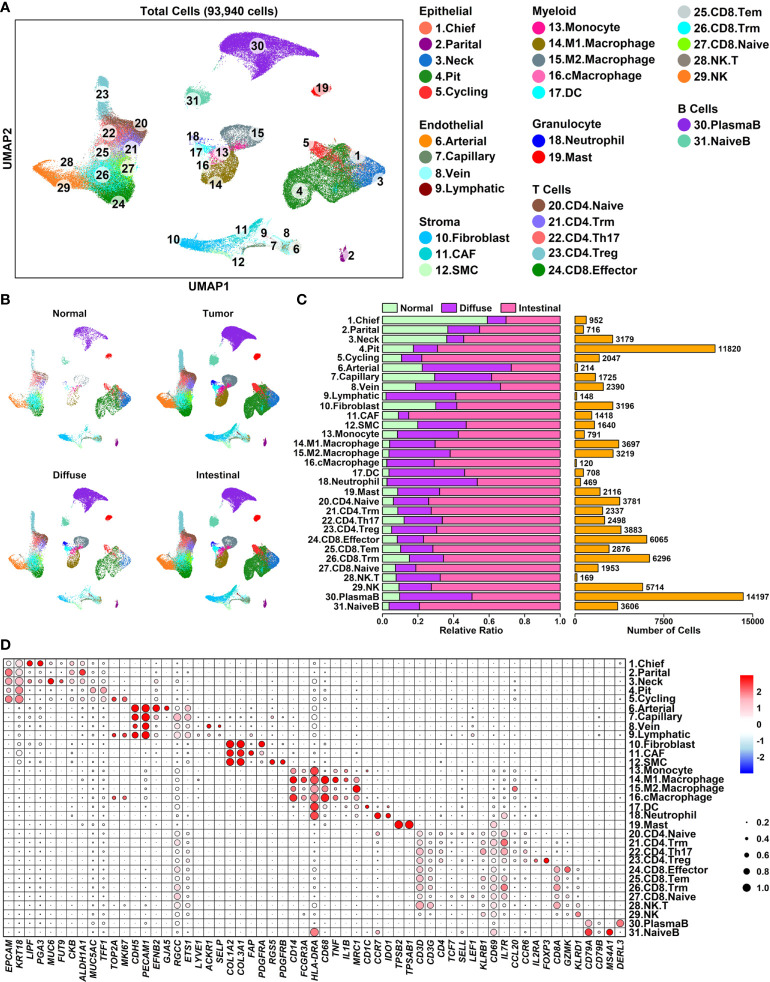
A single-cell atlas of human diffuse and intestinal type GC samples **(A)** UMAP plot showing the distribution patterns of 6 major groups and 25 subgroups; **(B)** UMAP plots showing the distribution patterns of all groups per sample type; **(C)** Bar plot showing the basic information of 25 subgroups; **(D)** Dot plot representing relative expression status of all marker genes used in the determination of each subgroup. Average expression values of each gene within a certain subgroup are represented using color, and the ratios of gene-expressing cells within a certain subgroup are represented using circle sizes.

To explore the expression status of these Pro and Con genes in different cell types of GC samples from a cellular perspective, we reconstructed a GC single-cell atlas containing 93,940 cells using downloaded scRNAseq data of 25 gastric samples (19) including 5 para cancer normal samples, 6 diffuse type samples, and 14 intestinal type samples (**Supplementary Table 4**, GSE183904), as shown in **Figure 4A**. Cells from different sample conditions are evenly distributed across all UMAP plots, suggesting the integration process of scRNAseq analysis is reliable (**Figure 4B**). These cells are further clustered into 7 major groups and 31 subgroups (**Figure 4C**) based on the relative expression status of their corresponding marker genes, as shown in **Figure 4D**. Specifically, Epithelial cells (18,714 cells, 19.92%) defined by cells expression *EPCAM/KRT18* are further clustered in to 5 subgroups: Chief cells (952 cells) marked by *LIPF/PGA3*, Parital cells (716 cells) marked by *CKB/ALDH1A1*, Neck mucus cells (Neck, 3,179 cells) marked by *MUC6/FUT9*, Pit mucus cells (Pit, 11,820 cells) marked by *MUC5A1/TFF1*, and Cycling cells (2,047 cells) marked by *TOP2A/MKI67*; Endothelial cells (4,477 cells, 4.77%) defined by cells expressing *CDH5/PECAM1* are further clustered into 4 subgroups: Arterial cells (214 cells) marked by *EFNB2/GJA5*, Capillary cells (1,725) cells marked by *RGCC/ETS1*, Vein cells (2,390 cells) marked by *ACKR1/SELP*, and Lymphatic vessel cells (Lymphatic, 148 cells) marked by *LYVE1*; Stroma cells (6,254 cells, 6.66%) defined by cells expressing *COL1A2/COL3A1* are further clustered into 3 subgroups: Fibroblast cells (3,196 cells) marked by *PDGFRB*, Cancer-associated fibroblasts (CAF, 1418 cells) marked by *RGS5*, Smooth muscle cells (SMC, 1,640 cells) marked by *FAP/PDGFRA*; Myeloid cells (8,641 cells, 9.20%) defined by cells expressing *HLA-DRA* are further clustered into 5 subgroups: Monocytes (791 cells) marked by *CD14/FCGR3A*, M1 polarized macrophages (M1.Macrophage, 3,697 cells) marked by *CD14/FCGR3A/CD68/TNF/IL1B*, M2 polarized macrophages (M2.Macropahge, 3,219 cells) marked by *CD14/FCGR3A/MRC1*, Cycling macrophages (cMacrophage, 120 cells) marked by *CD14/FCGR3A/CD68/TOP2A/MKI67*; Dendritic cells (DC, 708 cells) marked by *CD1C*; Granulocyte cells (2,479 cells) are further clustered into 2 subgroups: Neutrophil cells (469 cells) marked by CCR7/IDO1, Mast cells (2,116 cells) marked by TPSB2/TPSAB1; T cells (35,572 cells, 37.87%) defined by cells expressing *CD3D/CD3G* are further clustered into 10 subgroups: CD4 naïve T cells (CD4.Naive, 3,781 cells) marked by *CD4/TCF7/SELL/LEF1*, CD4 tissue resident memory T cells (CD4.Trm, 2,337 cells) marked by *CD4/IL7R/CD69*, CD4 helper 17 cells (CD4.Th17, 2,498 cells) marked by *CD4/CCL20/CCR6*, CD4 regulatory T cells (CD4.Treg, 3,883 cells) marked by *CD4/IL2RA/FOXP3*; CD8 effector T cells (CD8.Effector, 6,065 cells) marked by *CD8A/GZMK*, CD8 tissue effector memory T cells (CD8.Tem, 2,876 cells) marked by *CD8A/KLRB1*, CD8 tissue resident memory T cells (CD8.Trm, 6,296 cells) marked by *CD8A/CD69/IL7R*, CD8 naïve T cells (CD8.Naive, 1,953 cells) marked by *CD8A/TCF7/SELL/LEF1*, Natural killer T cells (NK.T, 169 cells) marked by *CD8A/KLRD1*, Natural killer cells (NK, 5,714 cells) marked by *KLRD1*; B cells defined by cells (17,803 cells, 18.95%) expressing *CD79A/CD79B* are further clustered into 2 subgroups: Plasma B cells (PlasmaB, 14,197 cells) marked by *DERL3*, Naïve B cells (NaiveB, 3,606 cells) marked by *MS4A1*.

### Expression status of pro and con genes in different cell types of GC samples

**Figure 5:**
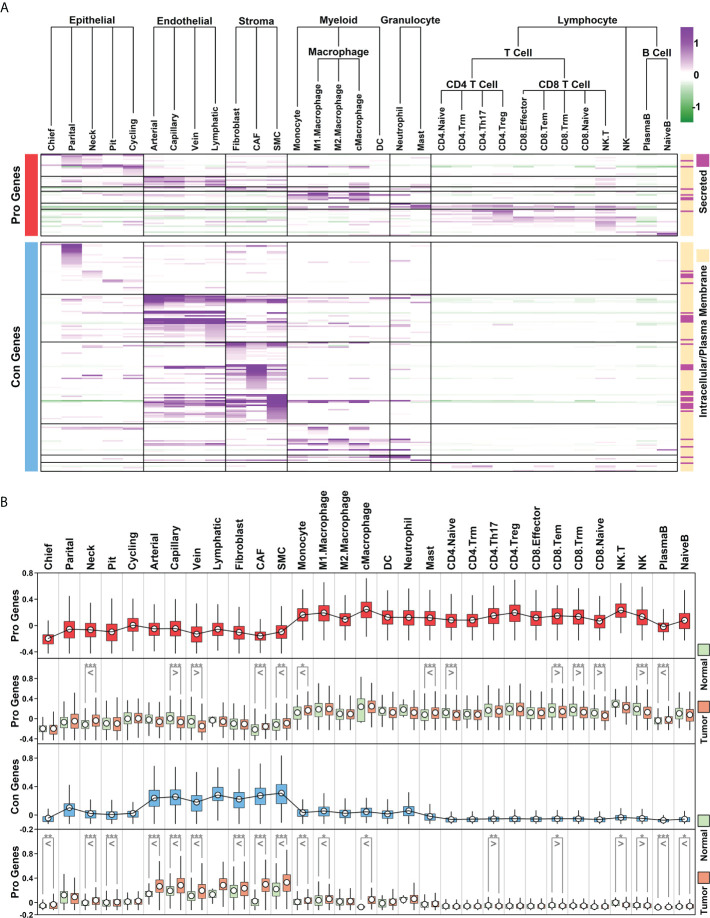
Cellular expression profiles of Pro and Con genes **(A)** Relative expression values of Pro and Con genes in 31 subgroups. Average expression values of each gene within a certain subgroup are represented using color. The subcellular location information of each gene is indicated on the right; **(B)** Boxplot representing the relative expression values of Pro and Con gene cohort in different subgroups/conditions (*p < 0.05; **p < 0.01; ***p < 0.001. Two-tailed t-test).

To examine the expression status of Pro and Con genes in different cell types of GC samples, we summarized the expression profiles of 55 Pro genes and 154 Con genes across 31 subgroups, as illustrated in **Figure 5A** and detailed in **Supplemental Table 5**. The Pro and Con genes are ranked based on their average expression values in different clusters. Part of the Pro and Con genes are not involved in these profiles due to the lack of their expression readings in scRNAseq data. Among 55 Pro genes, 18 of them (32.73%) have the highest expression values in lymphocyte components, followed by 15 of them (27.27%) in epithelial components, 8 of them (14.55%) in myeloid components, 7 of them (12.73%) in endothelial components, 4 of them (7.27%) in granulocyte components and 3 of them (5.45%) in stroma components. The majority of the Pro genes have the highest expression values in immune-related components (28 out of 55, 54.55%), which is consistent with the GO enrichment results of Pro genes, where top enriched GO items are immune-related (**Figure 2E**). Among 154 Con genes, 56 of them (36.37%) have the highest expression values in stroma components, followed by 35 of them (22.73%) in epithelial components, 32 of them (20.78%) in endothelial components, 16 of them (10.39%) in myeloid components, 10 of them (6.49%) in granulocyte components and 5 of them (3.25%) in lymphocyte components. The majority of the Con genes have the highest expression values in ECM-related components (stroma and endothelial components, 88 out of 154, 57.14%), which is consistent with the GO enrichment results of Con genes, where top enriched GO items are ECM-related functions (**Figure 2F**).

We further examined the expression status of the Pro/Con cohort in 31 subgroups, and compared the difference between normal and tumor samples, as shown in **Figure 5B**. In general, relative expression values of the Pro cohort are higher in immune-related components (myeloid, granulocyte, T cells and B cells) compared to these in other components, and among certain immune-related components (CD4.Naive, CD8.Tem, CD8.Trm, CD8.Naive, NK), the expression values of the Pro cohort are lower in tumor samples compared to these in normal samples; relative expression values of the Con cohort are higher in ECM-related components (endothelial and stroma cells) compared to these in other components, and among most of the ECM-related components (except lymphatic vessel cells due to the limited number of cells), the expression values of the Con cohort are higher in tumor samples compared to these in normal samples. These results suggest that the Pro/Con cohort might be involved in tumor progression.

### Depleted ECM and enhanced immune process co-contribute to 5-FU-benefit responses in GC

**Figure 6:**
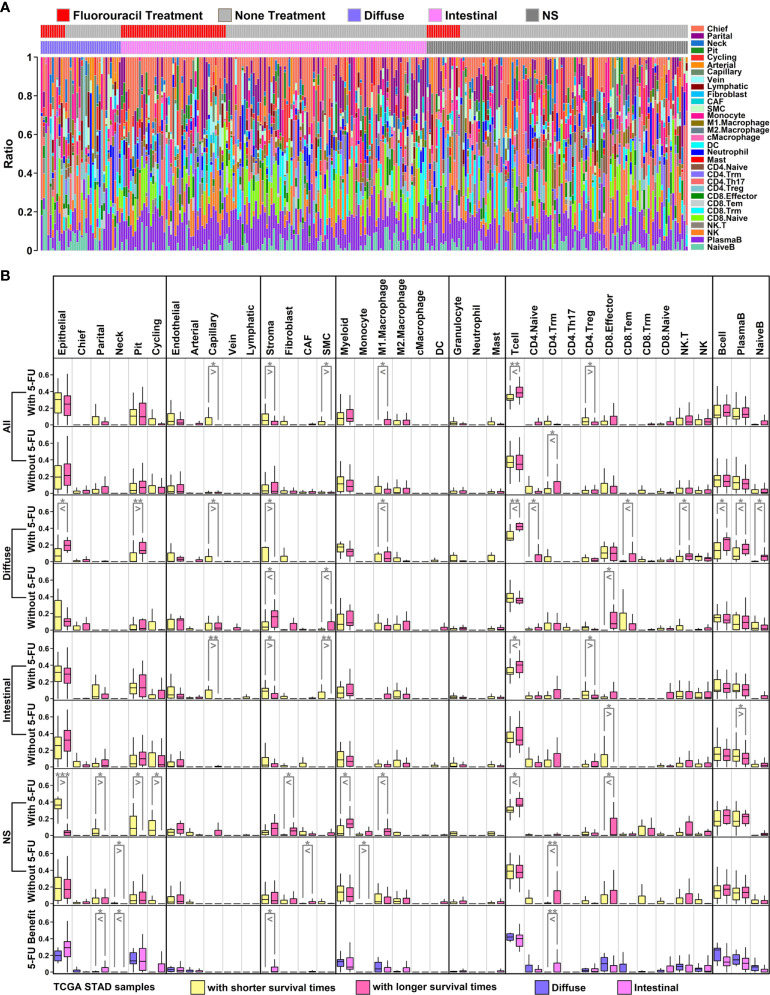
Proportion analysis of scRNAseq groups in TCGA STAD samples **(A)** Bar plot representing proportions of 31 subgroups in each TCGA sample. These data were generated through decomposition analysis; **(B)** Ratio comparison of 6 main groups/31 subgroups across different types of TCGA samples. All GC samples with 5-FU treatment: row 1; all GC samples without 5-FU treatment: row 2; diffuse samples with 5-FU treatment: row 3; diffuse samples without 5-FU treatment: row 4; intestinal samples with 5-FU treatment: row 5; intestinal samples without 5-FU treatment: row 6; NS samples with 5-FU treatment: row 7; NS samples without 5-FU treatment: row 8; diffuse 5-FU-benefit samples vs intestinal 5-FU-benefit samples: row 9. (*p < 0.05; **p < 0.01; ***p < 0.001. Two-tailed t-test).

To further explore the changes of ECM and immune components in GC samples with different 5-FU responses, we obtained cellular type proportion information of STAD samples through a deconvolution analysis (Bisque R package) (17) using RNA sequencing data and scRNAseq data (**Figure 6A**), and further analyzed the changes of these proportions among different groups (**Figure 6B**).

Among 6 major cell types, there is a significant decrease of stroma component, and a significant increase of T cell component in 5-FU-benefit GC samples (All types) compared to these in 5-FU-futile samples, while these significant changes are not examined in GC samples without 5-FU treatment (**Figure 6B**, first row and second row). Regarding 31 subgroups, there are significant decreases of capillary/SMC/CD4.Treg ratios, and significant increases of M1.Macrophage ratio in 5-FU-benefit GC samples (All types) compared to these in 5-FU-futile samples. Capillary and SMC cells are ECM-related components, and the reduction of these components in 5-FU-benefit samples indicates that an ECM-depletion process is beneficial for 5-FU treatment. M1.Macrophage cells are immune-promotive components while CD4.Treg cells are immune-suppressive component, and these changes suggests that an enhanced immune process is beneficial for 5-FU treatment.

Regarding specific GC subtypes, there are significant decreases of Capillary/Stroma ratios and increases of M1.Macrophage/Tcell/CD4.Naive/CD8.Tem/NK.T/Bcell/PlasmaB/NaiveB ratios in 5-FU-benefit diffuse samples compared to these in 5-FU-futile diffuse samples (**Figure 6B**, third row); there are significant decrease of Capillary/Stroma/SMC/CD4.Treg ratios and significant increases of Tcell ratio in 5-FU-benefit intestinal samples compared to these in 5-FU-futile intestinal samples (**Figure 6B**, fifth row). Interestingly, we find the stroma ratio is significantly lower in 5-FU-benefit diffuse samples compared to this in 5-FU-benefit intestinal samples, and the ratios of most immune components (Myeloid/M1.Macrophage/Tcell/CD8.Effector/CD8.Tem/NK.T/NK/Bcell/PlasmaB/NaiveB) are higher (although not significant) in 5-FU-benefit diffuse samples compared to these in 5-FU-benefit intestinal samples, suggesting a more depleted ECM and more enhanced immune process in 5-FU-benefit diffuse samples, which might explain the better performance of 5-FU on diffuse GC patients (**Table 1**).

### Key regulatory interactions in controlling 5-FU responses revealed by scRNAseq analysis

**Figure 7:**
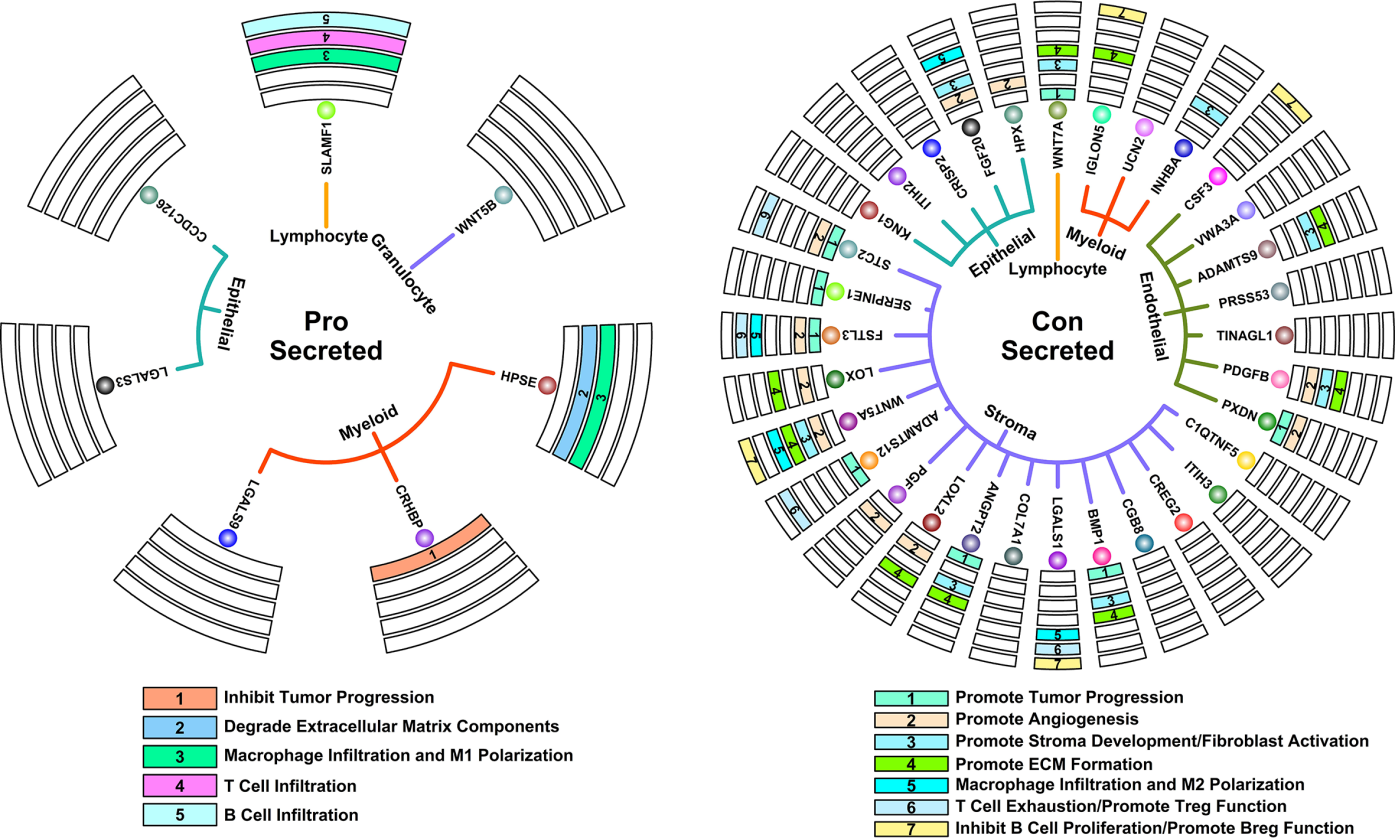
Key regulatory interactions in controlling 5-FU responses. Circle map representing secreted proteins that could contribute to 5-FU-benefit response (left); Circle map representing secreted proteins that could contribute to 5-FU-futile response (right).

A tumor is a complex system containing multiple components, and most of the time, these components do not work alone. Regulation by secreted proteins is one of the most direct means by which a single component can affect the entire TMEs. In this study, we further investigated possible regulation relationships between scRNAseq-defined cell types through the analysis of secreted proteins (**Figure 5A**, right panel, defined by subcellular location information from The Human Protein Atlas online database, www.proteinatlas.org) encoded by Pro and Con genes, as shown in **Figure 7**.

Among secreted proteins encoded by Pro genes, some of them have been previously confirmed of tumor inhibition ability in many studies. For example, CHRBP (Corticotropin releasing hormone binding protein) secreted by myeloid components could inhibit renal carcinoma progression (20); HPSE (Heparanase) secreted by myeloid components could promote the degradation of ECM, which further promotes the infiltration of T lymphocytes and hence antitumor ability (21); SLAMF1 (Signaling lymphocytic activation molecule family member 1) secreted by lymphocyte components could promote M1 macrophage anti-tumor polarization (22); SLAMF1 could also promote the infiltration and activation of certain T and B lymphocytes (23, 24). All these secreted proteins co-contribute to a tumor-suppressive microenvironment.

Among secreted proteins encoded Con genes, many of them have been previously confirmed of tumor promotion ability in many studies. For example, STC2 (Stanniocalcin 2), SERPINE1 (Serpin family E member 1), FSTL3 (Follistatin like 3), ADAMTS12 (ADAM metallopeptidase with thrombospondin type 1 motif 12), ANGPT2 (Angiopoietin 2), BMP1 (Bone morphogenetic protein 1) secreted by stroma components, PXDN (Peroxidasin) secreted by endothelial components and WNT7A (Wnt family member 7A) secreted by lymphocyte components could promote tumor progression/metastasis in many cancers (25–32); HPX (Hemopexin), FGF20 (Fibroblast growth factor 20) secreted by epithelial components, STC2, FSTL3, LOX (Lysyl oxidase), WNT5A (Wnt family member 5A), PGF (Placental growth factor), LOXL2 (Lysyl oxidase like 2) secreted by stroma components, PXDN, PDGFB (Platelet derived growth factor subunit B) secreted by myeloid components could promote angiogenesis process in many cancers (27, 33–41); FGF20 secreted by epithelial components, WNT5A and BMP1 secreted by stroma components, PDGFB and ADAMTS9 (ADAM metallopeptidase with thrombospondin type 1 motif 9) secreted by myeloid components could promote stroma development and fibroblast activations in many cancers (42–47); LOX, WNT5A, LOXL2, BMP1 secreted by stroma components, PDGFB and ADAMTS9 secreted by endothelial components, IGLON5 (IgLON family member 5) secreted by myeloid components, WNT7A secreted by Lymphocyte components could promote ECM formation (39, 43, 45–50); FGF20 secreted by epithelial components, FSTL3, WNT5A and LGALS1 (Galectin 1) secreted by stroma components could promote M2 macrophage pro-tumor polarization (51–54); STC2, FSTL3, ADAMTS12 and LGALS1 secreted by stroma components could promote T cell exhaustion process as well as promote immunosuppressive functions of regulatory T cells (28, 55–57); WNT5A, LGALS1 secreted by stroma components, CSF3 (Colony stimulating factor 3) secreted by endothelial components, IGLON5 secreted by myeloid components could promote infiltration and differentiation of regulatory B cell, as well as inhibit proliferation of B cells (58–61). All these secreted proteins co-contribute to a tumor-promoting microenvironment.

### Depleted ECM and infiltrated immune components in 5-FU-benefit patients validated by immunostaining experiments

**Figure 8:**
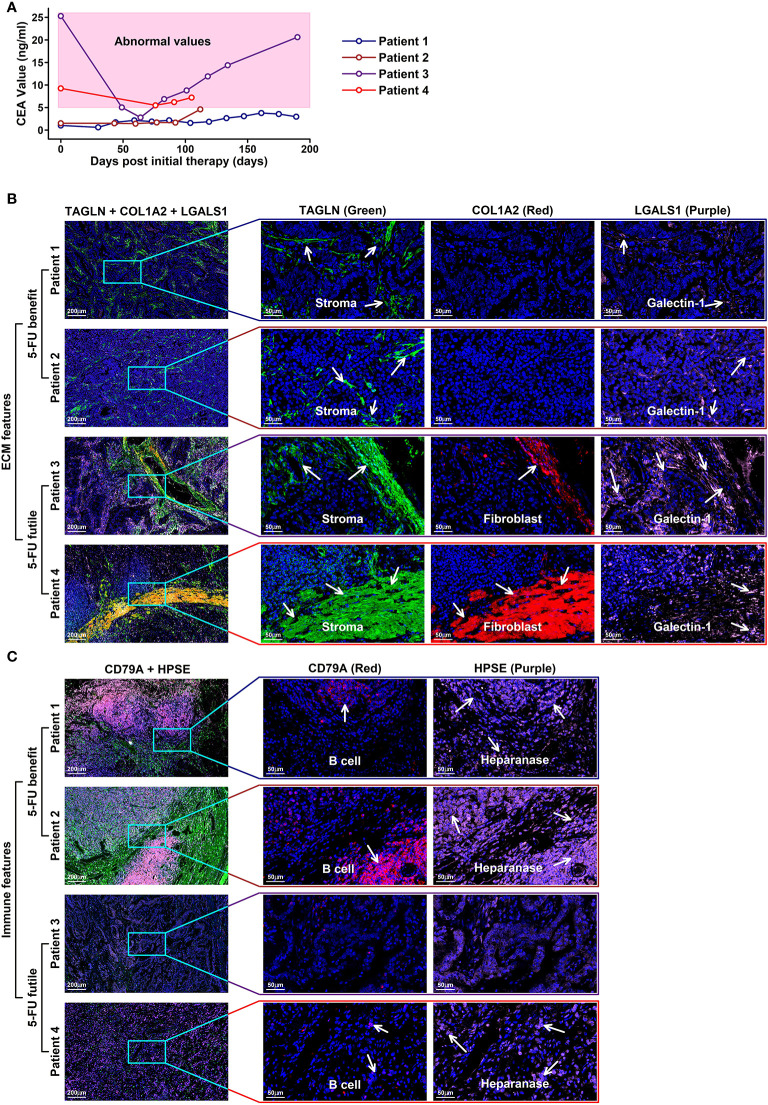
Immunostaining validation of TME features in 5-FU treated GC patients **(A)** CEA values of four GC patients with 5-FU chemotherapy treatments after radical gastrectomy; **(B)** ECM features in the tumor tissues of these four patients. Stroma cells are colored in green (TAGLN) and red (COL1A2); Galectin-1 is colored in purple (LGALS1). In 5-FU-benefit GC patients, more stroma cells as well as cells secreting galactin-1 are examined compared to those in 5-FU-futile GC patients. **(C)** Immune features in the tumor sections of these four patients. B cells are colored in red (CD79A); heparanase is colored in purple. In 5-FU-benefit GC patients, more B cells, as well as cells secreting heparanase are examined compared to those in 5-FU-futile GC patients.

To validate the TME features relating to different 5-FU responses, we involved four GC patients receiving 5-FU chemotherapy treatment (in combination with oxaliplatin and calcium folinate) after radical gastrectomy (clinical information listed in **Table 2**). Among these four patients, two of them (patient 1 and patient 2) have CEA (carcinoembryonic antigen) values less than 5 ng/ml, as revealed in the following serum tumor biomarker examinations, and these two patients are classified as 5-FU-benefit, while the other two patients (patient 3 and patient 4) with CEA values over 5 ng/ml (abnormal values), are classified as 5-FU-futile (**Figure 8A**). We further examined the specific TME component changes in these four patients using immunostaining of patient-specific tumor sections (**Figures 8B, C**). Regarding ECM components, there is an obvious depletion of stroma cells (marked by TAGLN, green) and fibroblasts (marked by COL1A2, red) in 5-FU-benefit patients, as well as an increased expression of Heparanase (HPSE, purple) which promotes the degradation of ECM. Regarding immune components, there is an obvious enrichment of B cells (marked by CD79A, red) in 5-FU-benefit patients, as well as a decreased expression of Galectin-1 (LGALS1, purple). Galectin-1 could promote an immune-suppressive environment (**Figure 7**), and the decrease of Galectin-1 facilitates the immune infiltration process in 5-FU-benefit patients. All these results confirm that the depletion of ECM and infiltration of immune components co-contribute to 5-FU-benefit responses in GC patients.

**Table 2:** Clinical information of involved GC patients.

Gastric Cancer	Patient 1	Patient 2	Patient 3	Patient 4
**Sex**	Male	Male	Male	Male
**Age**	67	66	68	70
**Histological Type**	Adenocarcinoma	Adenocarcinoma	Adenocarcinoma	Adenocarcinoma
**Lauren Type**	Diffuse	Diffuse	Intestinal	Diffuse
**TNM Stage**	pT3N2M0	pT4N2Mx	pT3N1M0	pT4N3M0
**Infiltration Degree**	pT3	pT4	T3	Pt4
**Tumor Stage**	IIIB	IIIB	IIB	IVA
**Radical Gastrectomy**	Yes	Yes	Yes	Yes
**Chemotherapy Plan**	Oxaliplatin (140mg) Calcium folinate (600mg) 5FU 0.6g dl, 4.0g CIV 48H	Oxaliplatin (120mg) Calcium folinate (590mg) 5FU 0.59g dl, 3.5g CIV 48H	Oxaliplatin (130mg) Calcium folinate (600mg) 5FU 0.6g dl, 4.0g CIV 48H	Oxaliplatin (100mg) Calcium folinate (500mg) 5FU 0.5g dl, 3.0g CIV 48H

## Discussion

5-FU has remained the most extensively used chemotherapeutic drug in the treatment of many cancers. Drug resistance to 5-FU is a common phenomenon in cancer chemotherapy. So far most of the studies exploring 5-FU-response predictions involved only 5-FU pathway-related genes/enzymes (6, 7) or certain types of factors such as immunohistochemical results (62) or inflammation (63), however, the tumor is a complex system comprising of ECM, stroma, vasculature and infiltrated immune cells other than tumor cells alone, and all these components have the potential to affect drug responses. Algorithms considering only one component would lead to biased estimation, hence in this study, we screened 5-FU-response-related genes transcriptome-wide and established a Pro and Con gene panel that could predict 5-FU response in different subtypes of GC patients. We further investigated the pan-cancer-wide applicability of this panel. The initial motivation of this pan-cancer exploration is to investigate which drug/cancer combination shares similar features with 5-FU/GC responses. For example, the Pro/Con cohort has an AUC score of 0.84 on Carboplatin/LUAD responses. We can reasonably speculate that 5-FU/STAD and Carboplatin/LUAD responses might share similar drug response mechanisms since the expression patterns of these Pro/Con genes have similarities. Based on these results, we believe that more insights would be inspired in future cancer therapy explorations.

Enriched ECM component or Desmoplasia has been considered as one of the main reasons causing anti-cancer drug resistance (64, 65), as these stiffened extracellular matrices could impede the infiltration of drugs and immune cells into the tumor. Besides, angiogenesis is often induced by hypoxia accompanying this stiffness process, in which disorganized neovascular vessels further lead to a decrease in drug delivery efficiency (66). In our results, we find that Con genes (high expression unfavoring 5-FU-benefit GC samples) are enriched in ECM-related functions (**Figure 2F**), and these genes are highly expressed in stroma/endothelial components (**Figures 5A, B**). We also observe a significant decrease in ratios of ECM-related components such as stroma/Capillary cells in 5-FU-benefit GC samples (**Figure 6B**) as well as depletion of ECM components in tumor sections of 5-FU-benefit GC patients (**Figure 8B**), confirming the importance of desmoplasia in mediating 5-FU responses. Based on our analysis and previous publications, the ECM depletion process (degradation of ECM components) in 5-FU-benefit GC patients could be possibly regulated through TME secreted protein HPSE, and the ECM enrichment process in 5-FU-futile patients could be possibly mediated through TME secreted proteins including LOX, WNT5A, LOXL2, ANGPT2, BMP1, PDGFB, ADAMTS9, IGLON5 and WNT7A (**Figure 7**).

Besides ECM remodeling, TIMEs (tumor immune microenvironments) are also reshaped during cancer progression and chemotherapy process, and these immune changes might also contribute to 5-FU responses in GC patients. In our study, we find that Pro genes (high expression favoring 5-FU-benefit GC samples) are enriched in immune-related functions (**Figure 2E****)**, and these genes are highly expressed in immune (myeloid, granulocyte and lymphocyte) components (**Figures 5A, B**), indicating an immune infiltration process in 5-FU-benefit GC samples. We also observe an increase of M1.Macrophage/Tcell/Bcell ratio in 5-FU-benefit GC samples (**Figure 6B**) as well as enrichment of B cells in tumor sections of 5-FU-benefit GC patients (**Figure 8B**), confirming the importance of immune components in mediating 5-FU responses. Macrophages have been demonstrated of great importance in mediating chemoresistance through M2 polarization in many cancers (67, 68). In our results, we find overexpression of secreted proteins including HPSE and SLAMF in TMEs of 5-FU-benefit samples that could induce an M1 macrophage polarization, and overexpression of secreted proteins including FGF20, FSTL3, WNT5A and LGALS1 in TMEs of 5-FU-futile samples that could induce an M2 macrophage polarization, suggesting that polarizations of macrophages are related to different 5-FU responses. Besides macrophages, we also find overexpression of secreted protein SLAMF1 in TMEs of 5-FU-benefit samples that could promote a T/B cell infiltration process, as well as overexpression of secreted proteins including STC2, FSTL3, WNT5A, ADAMTS12, LGALS1, CSF3 and IGLON5 in TMEs of 5-FU-futile samples that could promote a Treg/Breg infiltration and lymphocyte exhaustion process, confirming that lymphocytes infiltration/depletion are also related to 5-FU responses.

In this study, based on the combined analysis of bulk sequencing data and scRNAseq data, we found that depleted ECM components and enhanced immune process are two related features affecting 5-FU responses in GC, especially in diffuse GC patients. We also established a Pro and Con gene panel that could predict the 5-FU responses in GC patients, and proved partial applicability of this panel pan-cancer wide. Moreover, we further revealed possible regulatory mechanisms in these two processes (ECM and immune) based on scRNAseq data. Although this study is limited by the lack of scRNAseq data from patients with different 5-FU responses and a limited number of 5-FU-treated samples, still our results shed some new light on elucidating the mechanism of 5-FU resistance from TME perspectives, as well as provide potential therapeutic targets in overcoming this drug-resistance phenomenon.

## Data availability statement

The datasets presented in this study can be found in online repositories. The names of the repository/repositories and accession number(s) can be found in the article/**Supplementary Material**.

## Ethics statement

The studies involving human participants were reviewed and approved by ethical committee of Shenzhen People’s Hospital. The patients/participants provided their written informed consent to participate in this study. Written informed consent was obtained from the individual(s) for the publication of any potentially identifiable images or data included in this article.

## Author contributions

SD, HZ, JH, and CZ conceived the research idea. SD, HZ, and CZ prepared and wrote the manuscript. SD, SZ, and PZ performed data analysis. SZ, PZ, XM, JX, and JH collected the clinical samples. SZ and PZ performed immunostaining experiments. HZ, JH, and CZ revised the manuscript. All authors contributed to the article and approved the submitted version.

## Funding

This work was supported by the following: National Natural Science Foundation of China (32000465 to SD), China Postdoctoral Science Foundation 2020M680143 (to SD), China Postdoctoral Science Foundation 2021T140276 (to SD), the Guangdong Basic and Applied Basic Research Foundation (2020B1515120032, 2019B1515120033 to CZ), the Science and Technology Foundation of Shenzhen (JCYJ20210324115800001 to CZ), the Guangdong Provincial Natural Science Foundation (2021A1515010919 to CZ).

## Acknowledgments

We would like to thank the staffs of Department of Pathology in Shenzhen People’s Hospital for their technical support.

## Conflict of interest

The authors declare that the research was conducted in the absence of any commercial or financial relationships that could be construed as a potential conflict of interest.

## Publisher’s note

All claims expressed in this article are solely those of the authors and do not necessarily represent those of their affiliated organizations, or those of the publisher, the editors and the reviewers. Any product that may be evaluated in this article, or claim that may be made by its manufacturer, is not guaranteed or endorsed by the publisher.
